# New international guidance on quality, safety and efficacy of DNA vaccines

**DOI:** 10.1038/s41541-020-0199-0

**Published:** 2020-06-18

**Authors:** David W. C. Beasley

**Affiliations:** Department of Microbiology and Immunology, Institutional Office of Regulated Nonclinical Studies, Institute for Human Infections and Immunity, Sealy Institute for Vaccine Sciences, and WHO Collaborating Center for Vaccine Research, Evaluation and Training on Emerging Infectious Diseases, University of Texas Medical Branch, Galveston, TX USA

**Keywords:** DNA vaccines, Translational research, DNA vaccines

Over the past two decades, outbreaks of novel and re-emerging infectious diseases, particularly caused by zoonotic viruses, have prompted an international push to develop strategies addressing research, nonclinical and clinical testing, manufacturing, and regulatory evaluation that can increase the rapidity of medical countermeasure development to control outbreaks and prevent resurgence of those diseases. Most recently, the emergence of a novel coronavirus, SARS-CoV-2, and its explosive dissemination—resulting in a global disease pandemic (termed “coronavirus disease 2019”, or COVID-19) that, as of late April 2020, is associated with almost 3.1 million confirmed infections and 220,000 deaths^1^—has prompted an unprecedented response from the scientific and public health communities to develop therapies and vaccines. The publicly stated timeframes of 12–18 months for availability of licensed vaccines targeted against SARS-CoV-2 are optimistic, but would have been considered fantastic even a decade ago.

In response to other recent global public health emergencies, such as the 2014–2015 Ebola virus epidemic in West Africa and the widespread emergence of Zika virus in 2015–2016, several national and international health policy and funding agencies have prepared lists of priority pathogens considered to have potential to cause pandemic disease, and promoted the development of platform technologies that can speed the production of new vaccines against those agents, or against a previously unidentified emerging pathogen, often termed “Disease X”.

One such group of platform technologies that is considered by many to be a key component of rapid vaccine development are nucleic acid vaccines, in which the genetic sequences for potentially immunogenic and protective antigens from the pathogen are delivered as DNA or RNA molecules. Compared with more established approaches for immunization using injection of live attenuated or inactivated pathogens, or protein subunits of those pathogens, nucleic acid-based approaches offer several advantages for rapid response including rapid adaptability, simpler manufacturing processes, enhanced physical stability, and robust safety^2,3^. In addition, they may benefit from simplified requirements for nonclinical safety evaluation when alternative novel sequences are inserted into a previously well characterized platform construct. Some questions remain regarding the immunogenicity of nucleic acid vaccines compared with other more traditional vaccine approaches, but significant efforts have been made to address those through optimization of vaccination routes and timing of doses, and the use of vehicles and devices that facilitate uptake of the nucleic acid by host cells, resulting in improved performance of this class of vaccines in large animal models and in clinical use^2,3^.

Concurrent with facilitating new approaches and technologies that may speed vaccine development, the WHO’s R&D Blueprint for action to prevent epidemics has identified alignment of regulatory expectations for the testing and evaluation of novel vaccine candidates as a key enabler for effective outbreak responses^4^. In 2005, WHO developed “Guidelines for assuring the quality and nonclinical safety evaluation of DNA vaccines”^5^ and, based on the subsequent progression of several DNA vaccines to clinical evaluation and increasing awareness of the potential utility of DNA vaccines in outbreak responses, initiated a process for revision of those guidelines in February, 2018 from which a draft was publicly disseminated in mid-2019 for comment^6^. The revised Guidelines focus on biologically manufactured bacterial plasmid DNA for use in humans, and address aspects related to control of manufacture and characterization, approaches to nonclinical and clinical testing, and information that may be required by national regulatory authorities for approval of clinical trials or licensure. The revised Guidelines are considered unlikely to be applicable to RNA vaccines, and development by WHO of separate document(s) covering RNA vaccines is in progress^6^. In this issue of *npj Vaccines*, Sheets et al.^7^ provide an overview of the revision process to date, report the outcomes from an Informal Consultation of subject matter experts held in December, 2019 to review the draft revisions and discuss public comments that had been received, and describe the timeline for further revision and subsequent approval and implementation of the new guidelines by the WHO Expert Committee on Biological Standardization during 2020.

This communication is timely, given the current global impacts of COVID-19 and the rapid development of a large number of candidate vaccines, including several based on DNA or other nucleic acid platforms. At the time of this report (late April, 2020), and only 2–3 months following initiation of their development, a candidate DNA vaccine (ClinicalTrials.gov identifier NCT04283461) and a candidate RNA vaccine (NCT04336410) are in Phase 1 clinical trials, and more than a dozen other nucleic acid vaccine candidates are in development^8^. For the two vaccines now in Phase 1, it seems that the rapid path to clinical testing was facilitated by both the short timelines for manufacturing of initial clinical lots and streamlined requirements for nonclinical safety testing based on prior nonclinical and clinical experience with those platforms^8,9^. Evaluation of vaccine efficacy in newly developed animal models for COVID-19 can be expected to proceed in parallel with clinical safety testing. Vaccine development against SARS-CoV-2 is benefiting from prior experience with other closely related emerging coronaviruses associated with severe acute respiratory syndrome (SARS) and Middle East Respiratory Syndrome (MERS). Currently there are no licensed vaccines for coronaviruses that cause respiratory diseases in humans, and induction of robust and durable protective immunity against infection by SARS-CoV-2 through vaccination may be challenging. Furthermore, the outcomes of nonclinical efficacy testing of some candidate vaccines for SARS and MERS suggests that another significant concern is the potential for immune potentiation of COVID-19 lung disease^9–12^. The anticipated demand for hundreds of millions of doses for SARS-CoV-2 vaccines and their use in a wide range of target populations, coupled with the likely limitations of manufacturing capacity and scalability for any single vaccine type, suggests that engagement and coordination of multiple international partners for development of a portfolio of licensed vaccines based on multiple platforms will ideally be needed^13^.

The current COVID-19 pandemic will provide nucleic acid vaccine platforms their greatest opportunity to demonstrate in practice their proposed value as key components in the rapid response to controlling global or regional emerging infectious disease threats. The availability of the revised WHO Guidelines related to DNA vaccines, currently as a draft and subsequently in a final form, should facilitate communications between vaccine developers and regulatory agencies as nucleic acid vaccine candidates progress towards possible licensure.
