# Publisher Correction: New international guidance on quality, safety and efficacy of DNA vaccines

**DOI:** 10.1038/s41541-020-00217-z

**Published:** 2020-07-15

**Authors:** David W. C. Beasley

**Affiliations:** Department of Microbiology and Immunology, Institutional Office of Regulated Nonclinical Studies, Institute for Human Infections and Immunity, Sealy Institute for Vaccine Sciences, and WHO Collaborating Center for Vaccine Research, Evaluation and Training on Emerging Infectious Diseases, University of Texas Medical Branch, Galveston, TX USA

**Keywords:** DNA vaccines, Translational research, DNA vaccines

Correction to: *npj Vaccines*10.1038/s41541-020-0199-0, published online 18 June 2020

A reference was mistakenly omitted from the original version of this Comment. This has now been corrected in both the HTML and PDF versions.

